# Supplementary material for: Binding of SARS-CoV-2 Fusion Peptide to Host Endosome and Plasma Membrane
Source: J Phys Chem B. 2021 Jul 13;125(28):7732–41. doi: 10.1021/acs.jpcb.1c04176 (PMC8311640; doi:10.1021/acs.jpcb.1c04176)
Supplement: Supplementary file 1 — jp1c04176_si_002.pdf [file jp1c04176_si_002.pdf]

Supporting Information: Binding of SARS-CoV-2 Fusion Peptide to Host Endosome and Plasma Membrane

Stefan L. Schaefer,[†] Hendrik Jung,[†] and Gerhard Hummer[*,†,‡]

†*Department of Theoretical Biophysics, Max Planck Institute of Biophysics, 60438 Frankfurt am Main, Germany*

‡*Institute of Biophysics, Goethe University Frankfurt, 60438 Frankfurt am Main, Germany*

E-mail: gerhard.hummer@biophys.mpg.de

TABLE OF CONTENTS

- Supporting Tables S1 - S6
- Supporting Figures S1 - S4
- Supporting Movie Legend

Supporting Tables

Table S1: Restraints used during energy minimization (EM) and equilibration (EQ) steps of the fusion peptide in aqueous solution. Values in units of $kJ\,mol^{-1}\,nm^{-2}$.

Step	Backbone	Sidechains	Dihedrals
EM	400	40	4
EQ	400	400	4

Table S2: Outer plasma membrane-like lipid membrane composition following Lorent et al.[S1]

Lipid	Acyl chains	Full name	Abundance %
CHOL		Cholesterol	40
PSM	18:1/16:0	N-palmitoyl-D-erythro-sphingosylphosphorylcholine	12
NSM	18:1/24:1	N-nervonoyl-D-oleoyl-sphingosylphosphorylcholine	9.3
LSM	18:1/24:0	N-lignoceroyl-D-oleoyl-sphingosylphosphorylcholine	8
PLPC	16:0/18:2	1-palmitoyl-2-linoleoyl-sn-glycero-3-phosphocholine	14.7
SOPC	18:0/18:1	1-stearoyl-2-oleoylphosphatidylcholine	6.7
PAPC	16:0/20:4	1-palmitoyl-2-arachidonoyl-glycero-3-phosphocholine	5.3
PLA20(PE)	18:0/20:4	1-O-stearoyl-2-O-arachidonoyl-glycero-3-phosphoethanolamine	2.7
SAPS	18:0/20:4	1-stearoyl-2-arachidonoyl-glycero-3-phosphoserine	1.3

Table S3: Late endosomal membrane composition.

Lipid	Acyl chains	Full name	Abundance %
CHOL		Cholesterol	27
PLPC	16:0/18:2	1-palmitoyl-2-linoleoyl-sn-glycero-3-phosphocholine	22
POPE	16:0/18:1	1-palmitoyl-2-oleoyl-glycero-3-phosphoethanolamine	16
BMP	18:1/18:1	Bis(monoacylglycero)phosphate	11.6
POPC	16:0/18:1	1-palmitoyl-2-oleoyl-glycero-3-phosphocholine	11
SSM	18:1/18:0	N-stearoyl-D-erythro-sphingosylphosphorylcholine	7.3
SAPI	18:0/20:4	1-stearoyl-2-arachidonoyl-sn-glycero-3-phosphoinositol	3.65
SAPS	18:0/20:4	1-stearoyl-2-arachidonoyl-sn-glycero-3-phospho-L-serine	1.45

Table S4: Restraints used during energy minimization (EM) and equilibration (EQ) steps 1 to 6 of the endosomal and outer plasma membrane systems in $\mathrm{kJ\,mol^{-1}\,nm^{-2}}$.

Step	Time [ns]	Timestep [fs]	Backbone	Sidechains	Lipids	Dihedrals
EM			4000	2000	1000	1000
1	0.125	1	4000	2000	1000	1000
2	0.125	1	2000	1000	400	400
3	0.125	1	1000	500	400	200
4	0.5	2	500	200	200	200
5	0.5	2	200	50	40	100
6	0.5	2	50	0	0	0

Table S5: Simulated times in NTH pulling simulations.

initial state	run no.	simulated time [ns]
shallow	1	135
shallow	2	127
shallow	3	140
deep	1	195
deep	2	194
deep	3	172

Table S6: Fusion peptide secondary-structure elements in membrane contact during initial membrane binding event that led to stable binding and in final trajectory frame (initial/final).

membrane system	run no.	NTH bound	AH2 bound	CTH bound
endosomal	1	✓/✓	✓/✓	✗/✓
endosomal	2	✓/✓	✗/✗	✓/✓
endosomal	3	✗/✓	✓/✗	✓/✗
endosomal	4	✓/✓	✗/✗	✓/✗
outer plasma	1	✗/✗	✗/✓	✓/✓
outer plasma	2	✗/✗	✗/✗	✗/✗
outer plasma	3	✗/✗	✓/✓	✓/✗
outer plasma	4	✗/✗	✓/✓	✓/✓

Supporting Figures

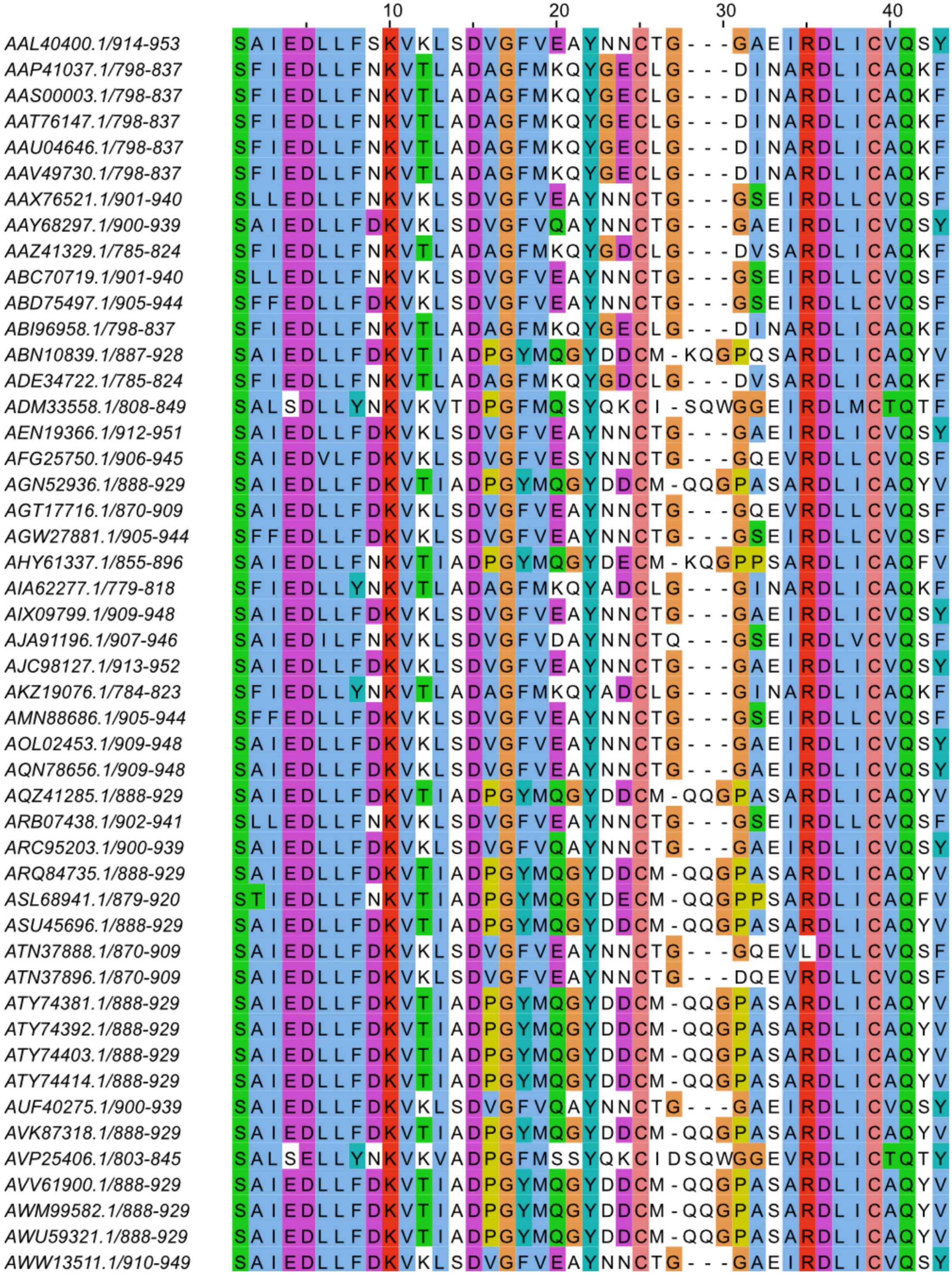

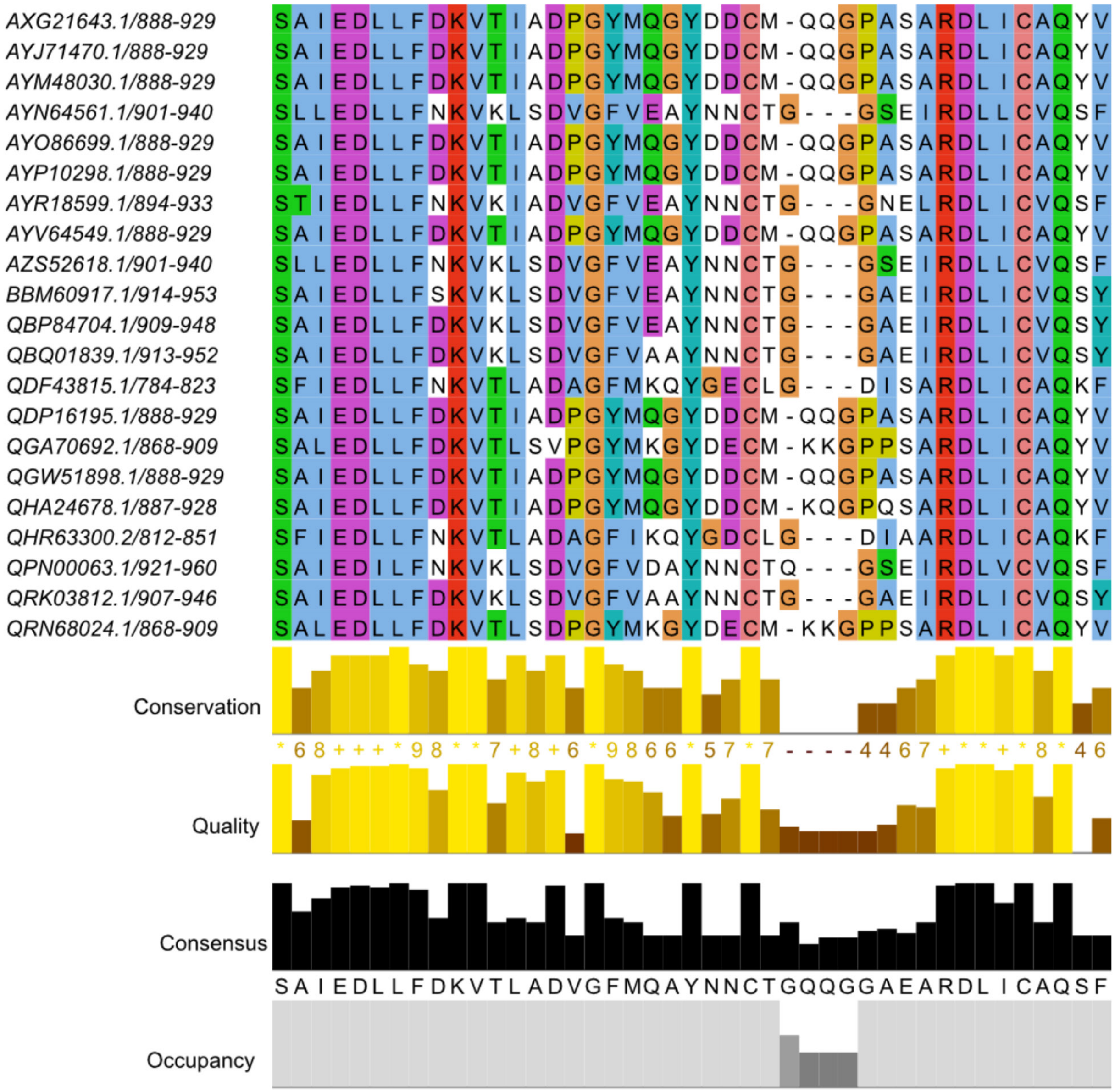

Figure S1: **Alignment of the fusion peptide region of betacoronaviruses.** Complete S protein sequences of the genus betacoronavirus were selected from the NCBI virus database[S2] and aligned using Clustal Omega.[S3] Only the fusion peptide region is shown. The fully conserved cysteines are at positions 25 and 39, respectively. Multiple entries from the same species were deleted. Coloring according to ClustalX.

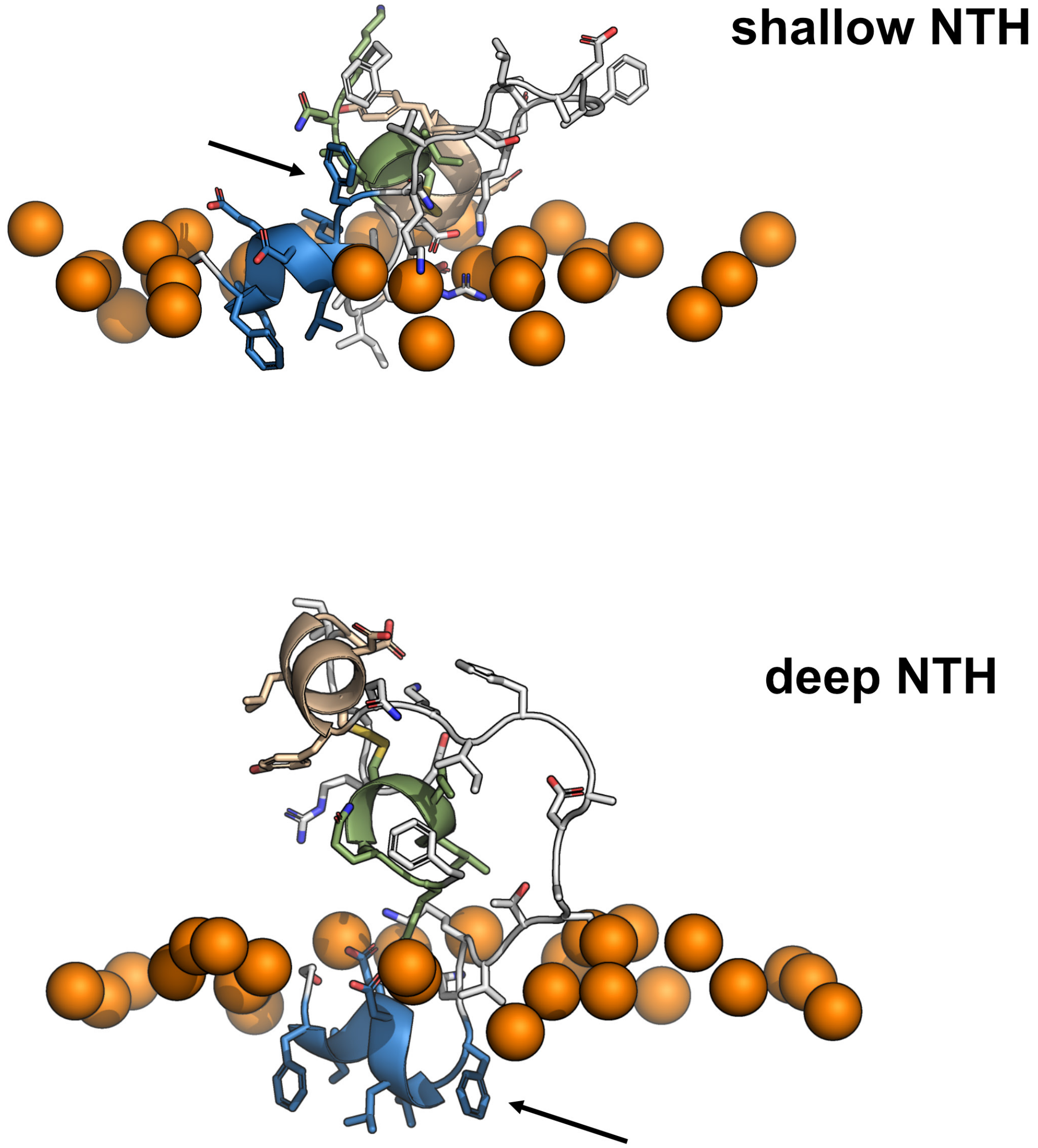

Figure S2: **Deep and shallow NTH insertion.** Arrows indicate the F823 position not inserted (top) and inserted (bottom) into the membrane interface. Colors as in Figure 1 (NTH: blue, AH2: beige, CTH: green). The upper membrane boundary is indicated by phosphate headgroups of nearby lipids (orange spheres).

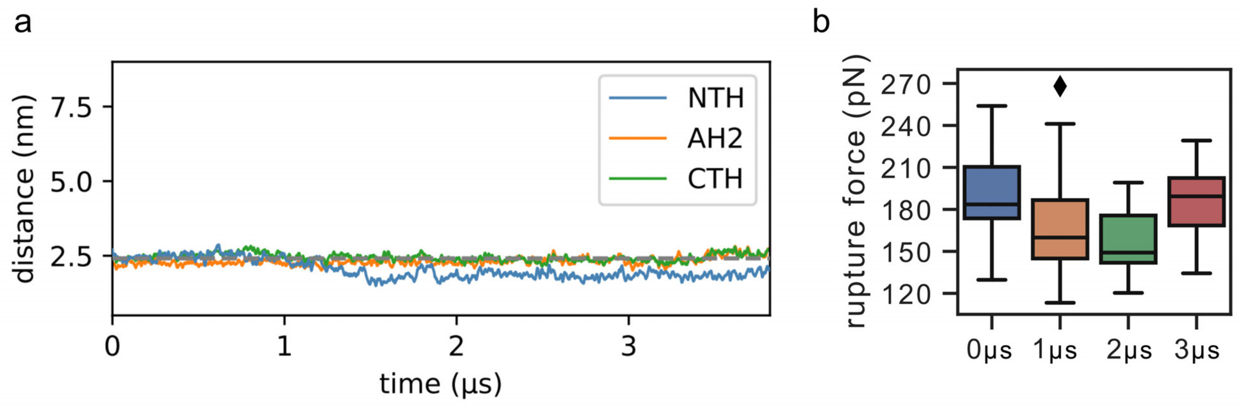

Figure S3: **Simulation starting with all three helices bound and adjusted outer-leaflet density.** (a) Distances of the centers of mass of the three helices and the center of mass of the membrane in which the lipid density of the outer leaflet was adjusted to accommodate the bound fusion peptide. The average phosphate position of the outer leaflet is indicated by a gray dotted line. (b) Box-plots showing the peak rupture forces of 20 replicate pulling simulations each for pulling simulations started with structures of the simulation taken at 0, 1, 2, and 3 μs (box: interquartile range; median: horizontal line; diamond: outlier).

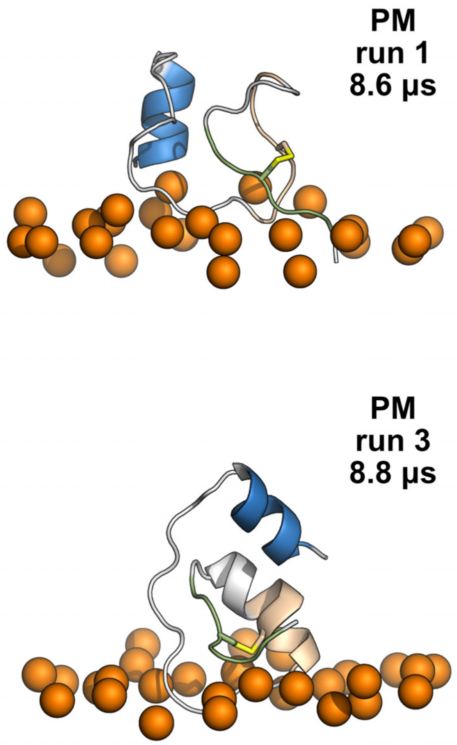

Figure S4: **Snapshots of MD simulations in which the AH2 and CTH partially unfolded to form flexible membrane bound structures.** (Left) Fusion peptide interactions with the mimetic of the outer plasma membrane (PM). (Right) Fusion peptide interactions with the mimetic of the endosomal membrane (EM). Time points are indicated. Colors as in Figure 1 (NTH: blue, AH2: beige, CTH: green). The upper membrane boundary is indicated by phosphate headgroups of nearby lipids (orange spheres).

Supporting Movie Legends

Supporting Movie S1

Dissociation of fusion peptide from membrane under mechanical force. (Left) Trajectory of the pulling simulation that resulted in the highest rupture force of about 250 pN. The fusion peptide is shown in the same colors as used in Figure 1. The gray sphere indicates the C-terminus, to which the pulling force is applied. Lipid headgroup phosphates are shown as yellow spheres. (Right) Force extension curve corresponding to the trajectory.

x-axis shows the distance of the C-terminal carbon (gray sphere) to the center of mass of the membrane. y-axis shows the force acting on the C-terminal carbon.

References

(S1) Lorent, J. H.; Levental, K. R.; Ganesan, L.; Rivera-Longsworth, G.; Sezgin, E.; Doktorova, M.; Lyman, E.; Levental, I. Plasma Membranes Are Asymmetric in Lipid Unsaturation, Packing and Protein Shape. *Nat. Chem. Biol.* **2020**, *16*, 644–652.

(S2) Hatcher, E. L.; Zhdanov, S. A.; Bao, Y.; Blinkova, O.; Nawrocki, E. P.; Ostapchuck, Y.; Schaffer, A. A.; Rodney Brister, J. Virus Variation Resource-Improved Response to Emergent Viral Outbreaks. *Nucleic Acids Res.* **2017**, *45*, D482–D490.

(S3) Madeira, F.; Park, Y. M.; Lee, J.; Buso, N.; Gur, T.; Madhusoodanan, N.; Basutkar, P.; Tivey, A. R.; Potter, S. C.; Finn, R. D.; Lopez, R. The EMBL-EBI Search and Sequence Analysis Tools APIs in 2019. *Nucleic Acids Res.* **2019**, *47*, W636–W641.
